# The application of a digital therapeutic reveals superior outcome compared to standard care in patients with anterior knee pain—a randomized controlled trial

**DOI:** 10.1007/s00402-025-05787-y

**Published:** 2025-03-12

**Authors:** Felix Ferner, Maximilian Schenke, Christoph Lutter, Wolf Petersen, Turgay Efe, Arno Schmeling, Kaywan Izadpanah, Florian Perwanger, Jörg Harrer, Jörg Dickschas

**Affiliations:** 1 Sana Kliniken Oberfranken, Lichtenfels, Germany; 2 OCO Schwandorf, Schwandorf, Germany; 3 Department of Orthopaedic Surgery, University Medical Center Rostock, Rostock, Germany; 4 Martin-Luther-Krankenhaus, Berlin, Germany; 5 Orthopaedicum Lich, Lich, Germany; 6 Sporthopaedicum Berlin, Berlin, Germany; 7 Department of Orthopaedic Surgery, University Medical Center Freiburg, Freiburg, Germany; 8 Orthoplus, Munich, Germany; 9 Sozialstiftung Bamberg, Bamberg, Germany; 10 Present Address: Department of Orthopaedic Surgery, University Medical Center, Erlangen, Germany; 11 Komitee Patellofemoral, Deutsche Kniegesellschaft, Schwarzenbek, Germany

**Keywords:** Anterior knee pain, Patellofemoral pain syndrome, Patellofemoral instability, Conservative treatment, Digital therapeutic

## Abstract

**Purpose:**

Anterior knee pain is a frequent symptom caused by disorders like patellofemoral pain syndrome or patella tendon tendinitis, which is commonly treated conservatively. The aim of the current study was to examine the effectiveness of a digital therapeutic (DT) versus German standard care through a prospective, randomized, multicenter controlled trial.

**Methods:**

Study participants were recruited in 9 orthopedic specialist centers diagnosed with anterior knee pain and a NPRS score of ≥ 4. Stratified randomization for n = 140 participants was conducted, assigning participants to the intervention group (exclusive use of DT) or the control group (standard care). Standard care included 1–3 prescriptions for physical therapy, while the DT consisted of a 90-day personalized exercise therapy program including educational content. The primary endpoints were pain measured by NPRS and functional impairment measured by the Kujala Score.

**Results:**

The use of the DT compared to the standard care showed an improvement in clinical outcomes (NPRS = − 3.7 ± 2.67 and Kujala = 18.00 ± 13.96, both p < 0.001). Both pain and function improved clinically and statistically significantly compared to the control group (ANOVA: NPRS = − 1.64 ± 0.35, p < 0.001, Kujala = 9.26 ± 2.52, p < 0.01).

**Conclusion:**

The use of the DT showed significant improvements in pain and function compared to the current standard therapy. These results are highly relevant for clinical practice in the treatment decision-making for patients with anterior knee pain as the DT bridges effectively gaps in the healthcare systems.

## Introduction

Anterior knee pain is a condition that particularly emerges in patients between 14 and 35 years of age [6]. Patients suffer from limitations in education, work, daily life, and leisure activities [20]. Anterior knee pain is most frequently induced by patellofemoral joint disorders such as patellofemoral pain syndrome (PFPS), patella tendon tendinitis (PTT) or cartilage defects of the patellofemoral joint. Patellofemoral joint disorders have a high prevalence, e.g., PFPS with a 1-year prevalence of 22.7% in an average 30-year-old sample of the UK general population [8, 23]. Incidence data are predominantly available for selected cohorts such as military personnel and athletes. Accordingly, the data show a high variability and range for PFPS, for example, from 22 to 1000 person-years in US military personnel between 18 and 25 years of age to 1080.5/1000 person-years for a mean 38-year-old novice hobby runner in Belgium [23]. In addition, disorders in the patellofemoral joint are associated with a high morbidity and chronification rate [5, 24]. After a differentiated diagnosis and exclusion of relevant risk factors for patellofemoral disorders a conservative therapy is recommended as the primary therapy for patients with anterior knee pain [4, 27]. This conservative treatment so far has included a modification of the physical activity and physiotherapy. Since Digital Therapeutics (DTx) have already proven benefits in the care of other medical specialties, such as psychiatric/psychotherapeutic treatment or addiction therapy, a rapid access to therapy and continuous, reproducible treatment procedures are ensured [2, 14, 19]. The authors considered the application of a DT program supported for this specific orthopedic indication. The rationale for the development of the DT companion patella (PrehApp GmbH, Erlangen, Germany) was to generate a medical benefit for patients suffering from anterior knee pain by providing a continuous, evidence-based digital exercise therapy individualised to the patients’ characteristics and outcomes. It was developed to create a new treatment component and a conservative DT for the affected patient group.

Thus, the aim of this study is to investigate the effectiveness of the DT compared to standard care as in person physiotherapy in patients with anterior knee pain in a prospective, multicenter, randomized, controlled study design. We hypothesize that the application of the DT combined with patient education program is superior to the so far “gold standard therapy” in patients with anterior knee pain in terms of pain reduction and functional outcome.

## Methods

### Study design

A randomized controlled trial was conducted: group A was treated with the self-guided DT application companion patella (PrehApp GmbH, Erlangen, Germany), whereas the control group B was treated with standard care. The DT group received a 90-day personalized exercise therapy program. Standard care consisted of physical therapy, which includes 1 to 3 prescriptions of 6 applications of physical therapy of 20–30 min respectively. All study-related data (e.g., baseline assessment, primary and secondary endpoint data) were collected between February 2022 and June 2023.

### Patient and public involvement

No members of the public or patients within conducting the trial and interpreting the findings were involved.

### Equity, diversity, and inclusion statement

The research team included 78 women and 58 men from rural and urban study centers in Germany. All nine study centers were specialized outpatient clinics for knee surgery and the surgeons were all certified knee surgeons (from the German Knee Society). The inclusion criteria were inclusive in regard of all socio-economic background, sexes, ethnicities/race, and marginalized groups (Table 1).

**Table 1:** Summary of inclusion and exclusion

Inclusion criteria	Exclusion criteria
Gender male or female Age 14 to 65 years NPRS ≥ 4 (within the last 24 h) One of the following diagnoses (According to the ICD classification): *Patellofemoral disease (M22.2)* *Chondromalacia patellae (M22.4)* *Tendinitis of the patella tendon (M76.5)* *Pain of the lower extremity (Fibula, tibia, knee) (M79.66)* *Luxation of the patella (S83.0)*	Missing patient agreement History of previous operations Recurrent patella dislocation Acute intraarticular pathologies (e.g., cartilage lesions, bone fractures) Relevant predisposing conditions for patellofemoral instability: *Dysplasia of the trochlea (type B, C or D according to Dejour)* *Torsional deformity of more than 10° or Q-angle* > *25° in clinical investigation* *Genu valgum* > *5°* Chronic or acute cardiac disease Dizziness Mental disability Pregnancy Conditions associated with deficient perception (e.g., medication) Incomplete language skills (German)

*NPRS* numeric pain rating scale, *ICD* international classification of diseases, 10. Revision, German Modification

### Participants

Patients that matched the inclusion and exclusion criteria were included in the study (Table 1). After confirming the diagnosis, the informed consent was given by all participants in written form. The baseline assessment was carried out by the treating physician at each study centers. The follow-up assessments were conducted by phone by the instructed personnel of the study center. Study participants did not receive any financial compensation for participating in the study.

### Intervention

**Fig. 1:**
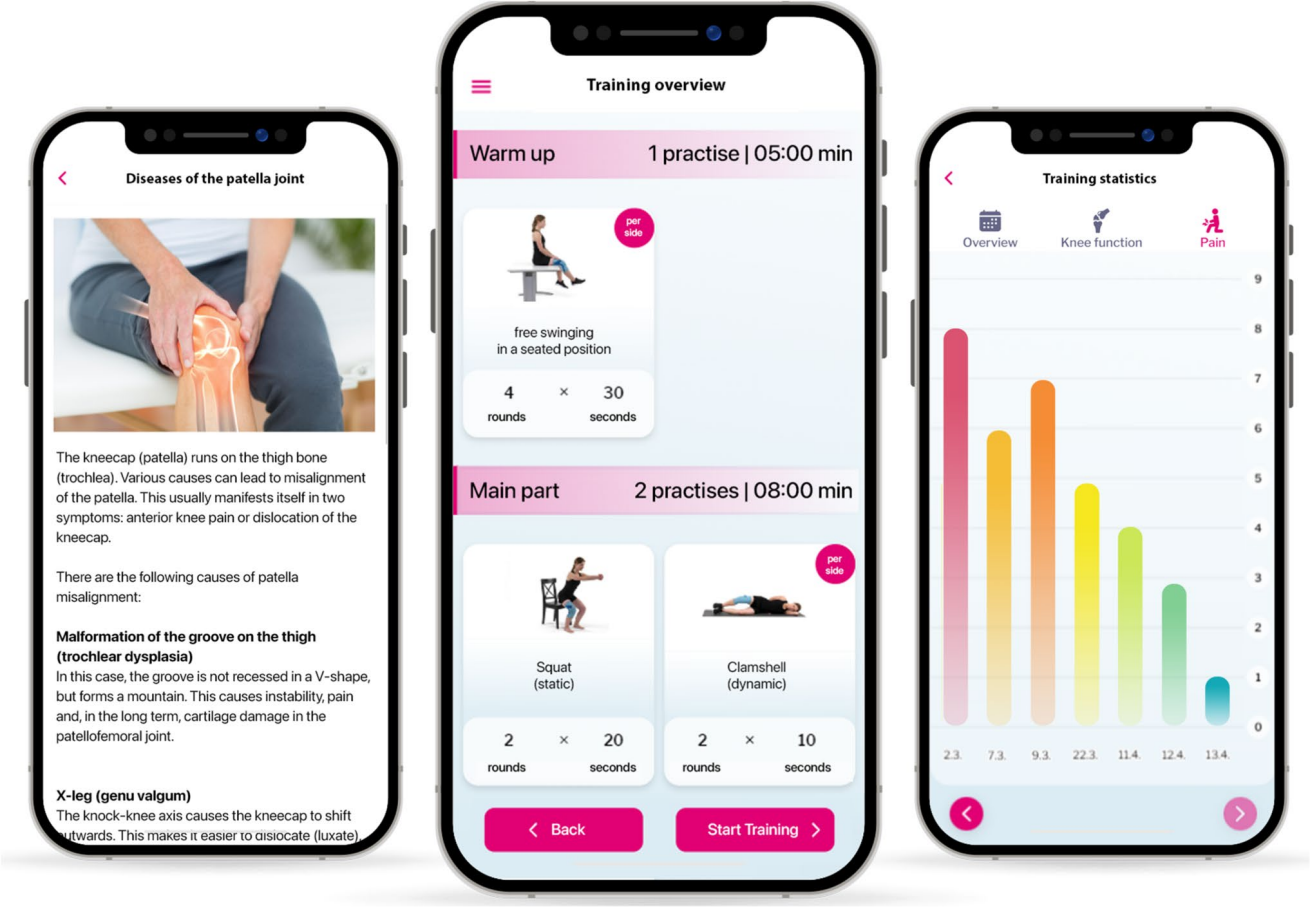
Screenshots from the DT companion patella demonstrate excerpts from the educational program (left), exercises (middle) and the statistics of the pain score over the time

Patients in the intervention group were provided access to the DT companion patella and encouraged by the clinical investigator to use the app on their smartphone or tablet at least three times a week, ideally daily, throughout the study duration of 90 days. The DT companion patella is a web application and was launched in March 2021. It is classified as a medical product class I according to the European medical device regulation (MDR). Companion patella  contains three main functions (1) exercise therapy, (2) education via library articles (3) therapy statistic section (Fig. 1A–C). The content in the educational library covers a broad spectrum of general illness/impairment and anterior knee pain-specific education in lay language. The single physiotherapeutic exercises and the individual composition of exercises are adjusted for every user. Exercises are ranked depending on exercise difficulty and strain. Depending on user feedback, exercises are constantly AI driven adapted to the user’s ability. In every single exercise a male or female model avatar initially demonstrates the correct execution and then the patient carries out the exercise simultaneously with the model supported by auditory assistance. The duration of the exercise sessions vary between 15 and 25 min. Special equipment is not required for the training, besides common household aids like a chair, bottle, stair, or room wall. The content for an individual patient is compiled and updated from day to day (or upon each login). Depending on the patient´s status of knowledge, practice, and progress this is adapted continually. Pain levels are recorded using a 11-point numeric pain rating scale (NPRS; 0 = no pain, 10 = unbearable pain) at the end of each therapy in a pain diary and the function is recorded using the Kujala Score and asked to be filled out by the patients every 4th week.

### Outcome measures

The following parameters were defined as primary endpoints: Pain in the affected knee joint, measured using the NPRS [3, 27], and function of the affected knee joint, measured by the Kujala score [7, 27]. The primary endpoints were queried in both groups at baseline in person by the investigator and 30, 60 and 90 days after admission by telephone by the study center. Adverse events occurring during the treatments were recorded as secondary endpoints. Patellar dislocations, muscle injuries and cardiovascular problems were defined as adverse events. Moreover, the use of pain medication was recorded. Data collection was identical for the intervention and control group. The duration of the treatment was 90 days and contained four measurement points. Participants received a version of the Kujala score as visual aid by email at the survey dates. If patients couldn’t be contacted, they were contacted again at the next survey time.

### Randomization and follow-up

Included participants were randomly allocated equally (allocation ratio 1:1) to the two groups. Randomization was stratified for each trial center and for the three main symptoms PFPS, PTS and patella dislocation. The randomization sequence was generated by the study center using a computer-controlled random number generator with MATLAB software (The MathWorks, Inc., Natick, USA). Patients and the treating physician only learned about the assignment to the intervention or control arm after the patient was included in the study. According to the assignment, the patient received either an access to the DT companion patella or physiotherapy according to the agreements with the statutory and private health insurance funds.

Both, patients and investigators were not blinded to reflect the actual care situation. The study nurse collecting the endpoints was not blinded. However, the biostatistician was blinded. The physiotherapists executing the control therapy were not involved in the study setting.

### Sample size

The sample size calculation was done using MATLAB software and was based on the following assumptions: Significance level α = 5%, Power 1 − β = 90%. Minimal clinically important difference (MCID) and standard deviation (SD) values of NPRS (MCID = − 1.16, SD = 2) and Kujala (MCID =  + 8–10, SD = 13). The calculation resulted in a total of 70 patients per group. Thus, 140 patients were recruited.

### Statistical analyses

The statistical analysis of the study data was performed after “Last Patient Out” and subsequent data consistency checks. Data were analysed using Software Version R 4.3.1 (2023-06-16 ucrt) Packages, according to the ITT-principle. Complete case analysis (CAA) and jump-to-reference (J2R) as one imputation method were carried out. Missing values at baseline were not imputed. The discussion of the results is based on the results from the J2R procedure. Primary outcome measures were analyzed by 2-by-2 (group-by-time, T0 and T3) mixed model analysis of variance (ANOVA) and Linear mixed model (LMM), separately for NPRS and for Kujala. The significance level was set to 5%. Additionally, a longitudinal “mixed model” was performed considering all measurements after randomization (T1, T2, T3) with the value at T0 as a covariate. In order to assess the significance of the group effect, two statistical analyses were carried out separately for NPRS and Kujala. For the J2R multiple imputations, a pooled effect with its t-statistic was additionally reported. All statistical tests were 2-sided. Moreover, a subgroup analysis for each indication (Patellofemoral Pain Syndrome = PFPS, Patella Tendon Tendinitis = PTT, Patellaluxation = LUX) was performed according to the same manner as it was used for the whole study population.

## Results

### Study population

The distribution of the demographic data is shown in Table 2. The two-tailed t-test analysis of the demographic data revealed no significant differences in age, height, weight and BMI between the intervention and control groups (Table 2). The risk ratio analysis (for dichotomous variables) and chi-square analysis (for dichotomous and categorical variables) revealed no significant differences in gender, distribution of indications, and use of orthoses between the two groups (Table 3).

**Table 2:** The continuous demographic data are summarized

	Intervention (N = 70) (MV ± SD)	Control (N = 66) (MV ± SD)	2-sided t-Test *p*
Age [years]	29.87 ± 11.81	28.78 ± 10.79	p = 0.57
Height [cm]	174.06 ± 9.52	173.18 ± 9.14	p = 0.59
Weight [kg]	73.41 ± 15.79	72.54 ± 15.49	p = 0.74
BMI [kg/m^2^]	24.15 ± 4.37	24.09 ± 4.39	p = 0.94

*MV* mean value, *SD* standard deviation

*p < 0.5; **p < 0.01; ***p < 0.001

**Table 3:** The categoric demographical data are summarized

	Intervention (N = 70)	Control (N = 66)	Risk-ratio p	Chi-squared Test p
Gender	Male 31 (44%) Female 39 (56%)	Male 27 (41%) Female 39 (59%)	p = 0.69 p = 0.69	p = 0.82
Indication	LUX: 8 (11%) PFPS: 37 (53%) PTT: 25 (36%)	LUX: 13 (20%) PFPS: 30 (45%) PTT: 23 (35%)	p = 0.19 p = 0.39 p = 0.91	p = 0.39
Knee side	Left 38 (54%) Right 32 (46%)	Left 41 (62%) Right 25 (38%)	p = 0.35 p = 0.36	p = 0.45
Use of orthosis	Yes 32 (46%) No 38 (54%)	Yes 28 (42%) No 38 (58%)	p = 0.70 p = 0.70	p = 0.83

*LUX* Luxation of the patella, *PFPS* patellofemoral pain syndrome, *PTT* patella tendon tendinitis

^*^p < 0.5; **p < 0.01; ***p < 0.001

The proportion of patients taking pain medication was not statistically significantly higher in the control group at baseline (p = 0.07) (Table 4). At T3, the number of missing values (n = 4) is low (≤ 5%) in both the intervention and control groups. A total of four patients withdrew their consent to participate in the study or to use their data for the purposes of the study (n = 3 intervention group, n = 1 control group). Ten patients were not reached at all time points.

**Table 4:** The distribution of pain medication intake is summarized

Pain medication n	Intervention	Control	Risk-ratio	Chi-squared test
T0	Yes 12 / 70 (17%)	Yes 21 / 66 (32%)	p = 0.05	p = 0.07
T3	Yes 4 / 69 (6%)	Yes 9 / 63 (14%)	p = 0.12	p = 0.18

*p < 0.05; **p < 0.01; ***p < 0.001

### Analysis of the primary endpoints

The baseline-analysis showed no significant differences in NPRS and Kujala between the intervention and control groups at baseline T0 (Table 5).

**Table 5:** The NPRS and Kujala Score and its p values are shown at T0

	Intervention	Control	Paired two-sided *t*-test *p*
N	70	66	
NPRS T0	5.97 ± 1.45	5.81 ± 1.36	p = 0.53
Kujala T0	69.06 ± 11.66	65.92 ± 15.86	p = 0.19

*MV* mean value, *SD* standard deviation

*p < 0.05; **p < 0.01; ***p < 0.001

**Fig. 2:**
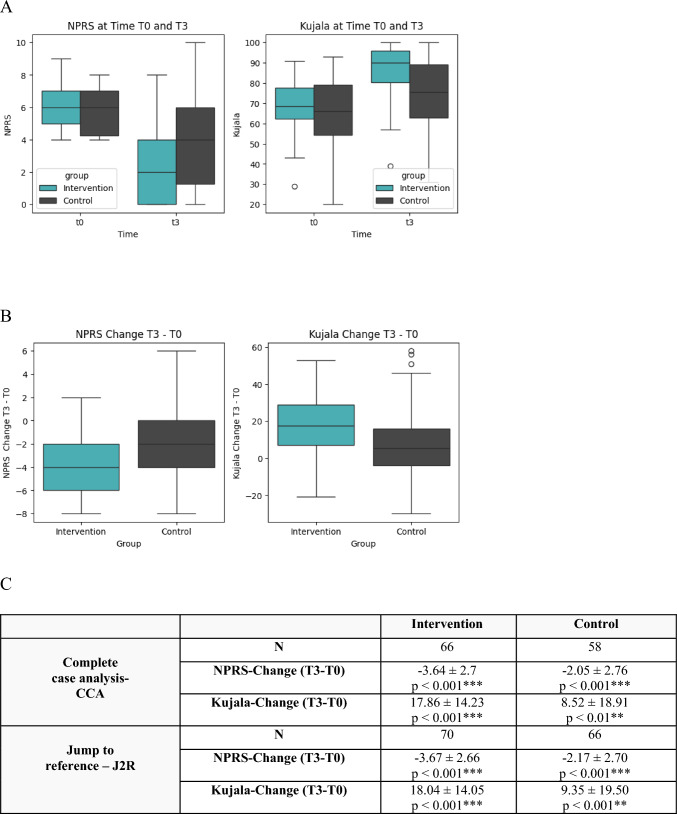
**A** The NPRS and Kujala score at T0 compared to T3 in both groups for the J2R method are shown. **B** The change in NPRS and Kujala score between T0 and T3 within both groups is calculated. **C** Additionally, the values for the change in NPRS and Kujala Score between T0 and T3 are shown for all cases together and one imputation method (J2R). *MV* mean value, *SD* standard deviation; *p < 0.05; **p < 0.01; ***p < 0.001)

To assess the significance of the change from baseline within each group, a two-tailed t-test analysis was performed for NPRS and Kujala respectively for each imputation method used. The analysis showed clinically (MCID- values: < − 1.16 points for NPRS and change > 8 points for Kujala) and statistically (p < 0.05) significant changes for both endpoints in both the intervention and control groups (Fig. 2C).

The data demonstrate a mean pain reduction of 62% within the intervention group. 17% of the patients (12/70) completed the study without pain or self-reported functional limitation (NPRS = 0, Kujala Score = 100). The control group showed a mean pain reduction of 36%. 8% of the patients (5/66) completed the study without pain or functional limitation (NPRS = 0, Kujala score = 100). The statistical analysis of the NPRS score and Kujala score shows a clinically (pain reduction effect < − 1.16 points, effect on Kujala > 8 points) and statistically significant (p < 0.05) effect for all imputation and statistical methods (Table 6).

**Table 6:** The ANOVA and LMM show a statistically significant effect measuring the NPRS and Kujala score

		NPRS	Kujala
Complete Case Analysis	N	Intervention: 66 Control: 58	Intervention: 66 Control: 58
	ANOVA	Effect (MV ± SE): − 1.59 ± 0.36 95% CI [− 2.29–0.88] p < 0.001*** Cohen’s d: − 0.64	Effect (M ± SE): 9.35 ± 2.53 95% CI [4.3414.35] p < 0.001*** Cohen’s d: 0.59
	LMM	Adopted Effect (MV ± SE): − 1.56 ± 0.40 95% CI [− 2.34–0.78] p < 0.001*** Cohen’s d: − 0.64	Adopted Effect (M ± SE): 9.55 ± 2.31 95% CI [4.9914.12] p < 0.001*** Cohen’s d: 0.64
Jump to reference	N	Intervention: 70 Control: 66	Intervention: 70 Control: 66
	Pooled Effect	Effect (MV ± SE): − 1.50 ± 0.35 95% CI [− 2.18–0.81] p < 0.001*** Cohen’s d: -0.60	Effect (M ± SE): 8.68 ± 2.52 95% CI [3.74 13.62] p < 0.001*** Cohen’s d: 0.54
	LMM	Adopted Effect (MV ± SE): − 1.45 ± 0.38 95% CI [− 2.31–0.60] p < 0.001*** Cohen’s d: − 0.59	Adopted Effect (M ± SE): 9.27 ± 2.28 95% CI [4.1114.42] p < 0.001*** Cohen’s d: 0.62

Analysis was performed for a complete case analysis and jump to reference imputation method with similar results

*MV* mean value, *SE* standard error, *CI* confidence interval

*p < 0.05; **p < 0.01; ***p < 0.001

**Fig. 3:**
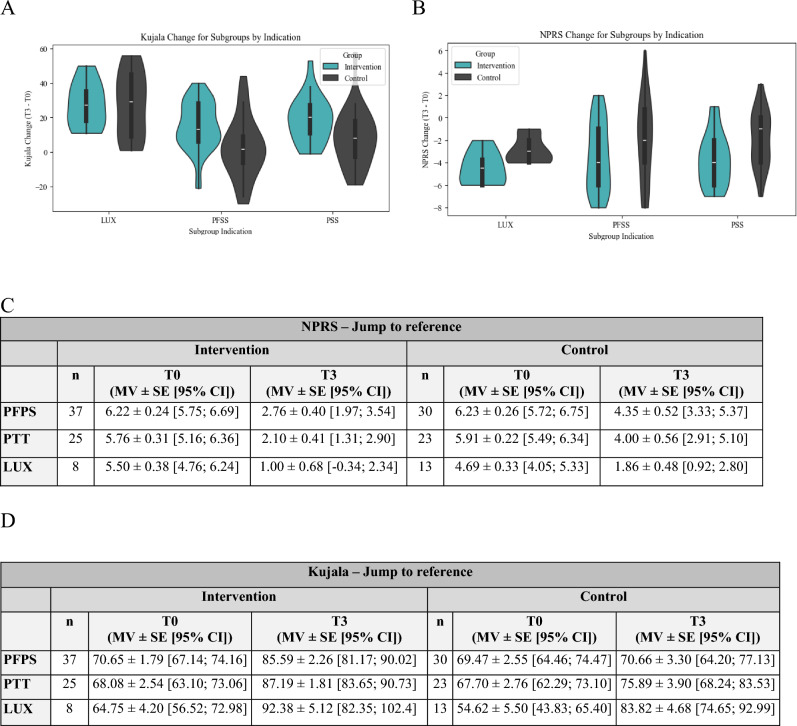
The violin plots depict the change of Kujala (**A**) and NPRS (**B**) separately for each subgroup (Lux, PFPS, PSS) of the intervention group compared to the control group. Hereby, the J2R imputation method was used. The exact values of the NPRS (**C**) and Kujala score (**D**) at T0 and T3 are shown divided into the three subgroups. *MV* mean value, *SE* standard error, *CI* confidence interval

The analysis of the subgroups revealed a statistically significant improvement in NRPS and Kujala for each subgroup (Fig. 3).

### Adverse effects and events

The sum of reported adverse effects in the intervention group is 2 (≙ 2.9%) and 0 (≙ 0%) in the control group: 0 ≙ 0%. One patient experienced increased pain after the second and third training session, whereupon the physician was contacted. After a 14-day grace period, therapy was resumed without further problems. One patient reported similar symptomatology at T2 and T3, namely that increased pain occurred after more intense exercise, whereupon the intervention was continued unabated.

### Use of pain medication

The percentage of patients taking pain medication was higher in the control group than in the intervention group throughout the study, although the difference was not statistically significant at either T0 or T3 (Table 4).

## Discussion

### Clinicial implications

For the first time, the effectiveness of a digital exercise program versus standard care was shown in patients with anterior knee pain regarding the outcomes pain and function in the German healthcare setting. Therefore, the hypothesis was proven correct. The results are of high relevance for the clinical practice in the therapy decision in patients with anterior knee pain. By using this new digital tool, the treatment of the patients suffering anterior knee pain can be lifted to a higher level compared to the established treatment without digital tools. Moreover, waiting periods up to three weeks for physiotherapy are reported in the German health care system as well as evidence practice gaps in musculoskeletal disorders [18]. Evidence about the actual utilization of conservative therapy in patients with anterior knee pain in Germany is sparse, as these indications are not part of the public health reporting.

The slightly different number of participants in the two groups (70 in the intervention, 66 in the control group) results from the randomization applied, which was stratified for each indication in the different trial centers. The average age of the two groups, 29.87 ± 11.81 years in the intervention group and 28.78 ± 10.79 years in the control group, is in line with the expected results in the literature for the indications studied [1, 15].

In both groups, women are slightly overrepresented with n = 39 (56%) in the intervention group and n = 39 (59%) in the control group, respectively. When considering the indications, the PFPS represents the largest proportion with 53% in the intervention group and 45% in the control group. Since PFPS mostly occurs in female patients, the slight overrepresentation of women included in the study may be explained [1, 17].

The analysis of the primary endpoints at T0 showed no significant differences in NPRS and Kujala between the intervention and control groups. The results at baseline are comparable to those reported in the literature. Fukuda et al., 2010. investigated different conventional physiotherapy approaches in a randomized study design and found baseline pain scores of 4.9 (Knee Exercise), 5.2 (Knee-Hip Exercise) and 4.9 (Control) on the NPRS scale in a comparison between 3 groups receiving knee-focused therapy alone (KE), knee- and hip-focused therapy (KHE) and a control group (CO) that did not receive physiotherapy. Similar values are documented by Saad et al., 2018, who showed in a randomized controlled trial with 4 groups (quadriceps strengthening, hip strengthening, stretching and control group without treatment) for the treatment of anterior knee pain. The authors found values between 4.03 and 6.34 on the NPRS scale at baseline. The results at baseline in the current study were slightly higher with with 5.97 ± 1.45 (NPRS) for the intervention group and 5.81 ± 1.36 (NPRS) in the control group. The values of the determined Kujala score at baseline show 69.06 ± 11.66 in the intervention group and 65.92 ± 15.86 in the control group. These values are comparable to the values detected by Fukuda et al., 2010 at baseline. The change in values from T0 to T3 was calculated with ‘Complete Case’ and two different imputation methods: Last observation carried forward and jump to reference method. Since the results showed no statistically significant difference because of the low number of drop-outs, the more conservative imputation method (J2R) was presented in the results part in order create a better clarity.

In the current study the control group received the physiotherapeutic treatment that has been “gold standard” in Germany to date according to the national guidelines [10] for the prescriptions of physiotherapy, which essentially corresponds to the interventions of the clinical studies known in the literature [9, 11, 21, 22]. In each case, physiotherapy treatments with a focus on the muscles around the knee or trunk stabilization are compared.

The improvement of − 2.06 ± 2.90 in the NPRS and 8.74 ± 19.41 in the Kujala score in the control group each represent a clinically relevant improvement (MCID NPRS = 1.16, Kujala = 8–10) and at the same time are within the range of data from different clinical studies. Hott et al. achieved a − 1.3-point improvement in pain and a + 8.8-point improvement in Kujala score in an intervention group with physiotherapy focused on hip and knee training [11]. In a systematic review, Manojlović et al. examined the data on the effects of a trunk-, hip- and knee-comprehensive training program on anterior knee pain [16]. It was shown here that the values of the control group are in the range of the values from the respective groups with a focus on pure knee therapy [16]. Various studies show values between − 1 and − 2.2 (NPRS) in pain improvement with knee-focused therapy [11, 12, 21, 22]. With the same therapy, the improvements in the Kujala Score range between + 6.7 and + 8.8. A broader therapy plan with additional exercises in the hip-torso area is somewhat higher in terms of pain and functional improvement, with pain improvement values between -1.3 and -3.3 and Kujala Score values between + 8.8 and + 14. The improvements in the intervention group of the current study are significantly higher (intervention group NPRS − 3.70 ± 2.67 and Kujala 18.00 ± 13.96).

Previous randomized controlled trials [25, 26] proved the use of DTx for different indications in the field of orthopedics. Toelle et al. and Weise et al. found significant improvements in pain scores when using their DTx Vivira (Vivira Health Lab, Berlin, Germany) and Kaia (Kaia health software GmbH, Munich, Germany)) in patients with lower back pain. The DT application Mawendo [13]*,* which also treats anterior knee pain, has so far only published the results of the systematic data collection. Here, a non-randomised study shows a mean improvement/reduction of 27 points on the Visual Analogue Scale (0–100) for pain and a mean improvement of 10 points in the Kujala score for the use of the digital application [13]. Again, the use of the DT companion patella achieves higher improvements in both endpoints, in a prospective randomised controlled trial design.

### Limitations

The number of cases in the different subgroups is relatively small. This can be explained by the initial study protocol, where a subgroup analysis was not planned. Nevertheless, a subgroup analysis for each indication was performed, and its results are presented in the current study. Further studies with a higher number of cases for each subgroup may be necessary in the future to receive more information about each specific subgroup. Another limitation may be that the patients of the control group did not receive a standardized homogeneous procedure. Since this procedure reflects the daily routine in health care system, the authors decided not to give the physiotherapists a standardized protocol.

## Conclusion

The use of the DT companion patella shows a clinically relevant as well as a statistically significant improvement in the measured endpoints (pain and function scores) compared to the previous standard of care with physiotherapy. Both pain and function improved clinically and statistically significant in patients with anterior knee pain compared to the previous standard of care. The treatment of the intervention group led to a complete reduction of symptoms in 17% of cases and resulted in a mean pain reduction of 62%. From the results, it can be concluded that the usage of the DT companion patella should be considered as part of the therapy algorithm for patients with anterior knee pain in everyday clinical practice.

## Data Availability

No datasets were generated or analysed during the current study.

## Abbreviations

PFPS: Patellofemoral pain syndrome
PTT: Patella tendon tendinitis
DT: Digital therapeutic
NRPS: Numeric pain rating scale
MCID: Minimal clinically important difference
SD: Standard deviation
SE: Standard error
CCA: Complete case analysis
LUX: Patellaluxation
J2R: Jump to reference
CI: Confidence interval
ICD: International classification of diseases
ANOVA: Analysis of variance
LMM: Linear mixed model
